# *Neurobiology of Language*: Volume 6 Reviewers List

**DOI:** 10.1162/NOL.x.234

**Published:** 2025-12-18

Steven, Meisler

Valentina, Borghesani

JeYoung, Jung

Hung-Shao, Cheng

Daniele, Gatti

Denise, Harvey

Lars, Meyer

Oscar, Woolnough

William, Matchin

Yingying, Wang

Xiangbin, Teng

Xing, Tian

Theodore, Turesky

Paul, Sowman

Peter, Turkeltaub

Qiming, Yuan

Ralf, Schmälzle

Robin, Gerrits

Rose, Bruffaerts

Salomi, Asaridou

Saloni, Krishnan

Simone, Gastaldon

Spencer, Kelly

Lars, Strother

Laura, Giglio

Jonathan, Brennan

Josh, Neudorf

Judit, Gervain

Jennifer, Krizman

Lisa, Goffman

Luigi, Grisoni

Mandy, Hampton Wray

Marc, Joanisse

Mathias, Scharinger

Matthias, Schlesewsky

Max, Coltheart

Maxime, Montembeault

Maya, Inbar

Meaghan, Perdue

Meredith, Shafto

Merve, Fritsch

Mikael, Roll

Nabin, Koirala

Neil, Cohn

Nilgoun, Bahar

Diana, López Barroso

Akshay, Maggu

Christian, Kell

Claude, Alain

Daniel, Lametti

Daniel, Llano

Alex, White

Alexander, Huth

Ana, Zappa

Andrea, Krott

Andrew, Bowers

Anna, Ivanova

Anna, Woollams

Cas, Coopmans

David, Copland

Erica, Flaten

Eunice, Fernandes

Fan, Cao

Eve, Higby

Gabriel, Cler

Giulio, Contemori

Hayoung, Song

Iring, Koch

Jacqueline, Cummine

Jamie, Reilly

Jason, Tourville

Jen-Kai, Chen

Jin, Wang

JingJing, Zhao

Jixing, Li

John, Gabrieli

Hiromu, Sakai

Guillaume, Herbet

Hannah, Rowe

Hyojin, Park

Ina, Bornkessel-Schlesewsky

Irene, Echeverria-Altuna

Gabriela, Vigliocco

Garikoitz, Lerma Usabiaga

F.-Xavier, Alario

Ferenc, Kemeny

Florencia, Assaneo

Francesca, Peressotti

Franck, Ramus

Fulvia, Palesi

Eva, Gutierrez-Sigut

Evan, Usler

Ethan, Roy

David, Jenson

David, McAlpine

Davide, Giampiccolo

Chad, Rogers

Chotiga, Pattamadilok

Chris, Rorden

Christian, Brodbeck

Anna-Lisa, Schuler

Anthony, Dick

Aurélie, Pistono

Benjamin, Parrell

Angela, Morgan

Angela, Pasqualotto

Anila, D'Mello

Cory, Shain

Christina, Zhao

Alec, Marantz

Dinglan, Tang

Donald, Bolger

Elizabeth, Hirshorn

Elizabeth, Schotter

Elliot, Murphy

Emily, Myers

Emmanuel, Mandonnet

Olaf, Hauk

Nai, Ding

Michal, Lavidor

Michele, Diaz

Maya, Yablonski

Matt, Davis

Marijn, van Vliet

Markus, Kiefer

Manuel, Perea

Lynn, Kurteff

Maaike, Vandermosten

Loïc, Labache

Jessica, Church

Junhua, Ding

Katherine, Travis

Kathleen, Gates

Kathrin, Rothermich

Linda, Romanovska

Stefan, Frank

Stephane, Dufau

Stephen, Nadeau

Steve, Majerus

Sivan, Jossinger

Soila, Kuuluvainen

Soo-Eun, Chang

Sara, Beach

Sarah, Tune

Satu, Saalasti

Shailee, Jain

Shalini, Narayana

Simon, Davis

Rutvik, Desai

Rocco, Chiou

Rashi, Pant

Rebecca, Jackson

Rebecca, Roth

Qing, Cai

Qingfang, Zhang

Qiuhai, Yue

Priyanka, Shah-Basak

P, Corina

Tuomo, Häikiö

Tzipi, Horowitz-Kraus

Uri, Hasson

Urs, Maurer

Vicky, Lai

Vitor, Zimmerer

Wei, Zhou

Wenjia, Zhang

Yang, Zhang

Yanni, Liu

Yi, Wei

Wonil, Choi

Talat, Bulut

Xiaoxia, Feng

Xiaoying, Wang

Yulia, Oganian

Ajay, Halai

